# 2-[(Phenyl­carbamo­yl)amino]­butyl *N*-phenyl­carbamate

**DOI:** 10.1107/S1600536812038081

**Published:** 2012-09-08

**Authors:** Shaaban K. Mohamed, Goran A. Bogdanović, Mehmet Akkurt, Antar A. Abdelhamid, Sabry H. H. Younes

**Affiliations:** a Chemistry and Environmental Division, Manchester Metropolitan University, Manchester M1 5GD, England; b ’Vinča’ Institute of Nuclear Sciences, Laboratory of Theoretical Physics and Condensed Matter Physics, University of Belgrade, PO Box 522, 11001 Belgrade, Serbia; c Department of Physics, Faculty of Sciences, Erciyes University, 38039 Kayseri, Turkey; d School Department of Chemistry, Faculty of Science, Sohag University, 82524 Sohag, Egypt

## Abstract

In the title compound, C_18_H_21_N_3_O_3_, the terminal phenyl rings make a dihedral angle of 86.3 (5)°. In the crystal, mol­ecules are linked by N—H⋯O hydrogen bonds into chains along [001], forming parallel *C*(4) and *R*_1_^2^(6) graph-set motifs.

## Related literature

For pharmaceutical properties of carbamates and carbamide compounds, see: Li *et al.* (2009 ▶); Gisbert & Pajares (2004 ▶); Metcalf (2002 ▶); Ray & Chaturvedi (2004 ▶). For a related structure, see: Ghalib *et al.* (2010 ▶). For graph-set motifs, see: Bernstein *et al.* (1995 ▶).
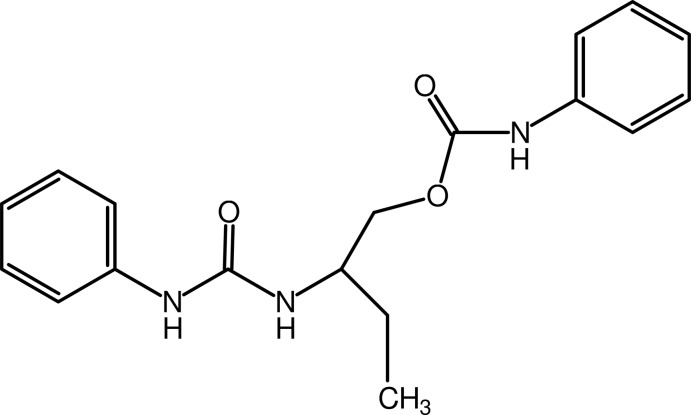


## Experimental

### 

#### Crystal data

C_18_H_21_N_3_O_3_
*M* *_r_* = 327.38
Monoclinic, 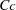

*a* = 10.722 (5) Å
*b* = 22.297 (3) Å
*c* = 9.109 (3) Å
β = 123.570 (6)°
*V* = 1814.5 (11) Å^3^
*Z* = 4
Cu *K*α radiation
μ = 0.68 mm^−1^
*T* = 293 K
0.14 × 0.12 × 0.07 mm

#### Data collection

Oxford Diffraction Xcalibur (Sapphire3, Gemini) diffractometer
2915 measured reflections
1676 independent reflections
1264 reflections with *I* > 2σ(*I*)
*R*_int_ = 0.024

#### Refinement

*R*[*F* ^2^ > 2σ(*F* ^2^)] = 0.052
*wR*(*F* ^2^) = 0.155
*S* = 1.05
1676 reflections
231 parameters
5 restraints
H atoms treated by a mixture of independent and constrained refinement
Δρ_max_ = 0.22 e Å^−3^
Δρ_min_ = −0.22 e Å^−3^

### 

Data collection: *CrysAlis PRO* (Oxford Diffraction, 2009 ▶); cell refinement: *CrysAlis PRO*; data reduction: *CrysAlis PRO*; program(s) used to solve structure: *SHELXS97* (Sheldrick, 2008 ▶); program(s) used to refine structure: *SHELXL97* (Sheldrick, 2008 ▶); molecular graphics: *ORTEP-3 for Windows* (Farrugia, 1997 ▶) and *PLATON* (Spek, 2009 ▶); software used to prepare material for publication: *WinGX* (Farrugia, 1999 ▶) and *PLATON*.

## Supplementary Material

Crystal structure: contains datablock(s) global, I. DOI: 10.1107/S1600536812038081/hg5248sup1.cif

Structure factors: contains datablock(s) I. DOI: 10.1107/S1600536812038081/hg5248Isup2.hkl

Supplementary material file. DOI: 10.1107/S1600536812038081/hg5248Isup3.cml

Additional supplementary materials:  crystallographic information; 3D view; checkCIF report

## Figures and Tables

**Table 1:** Hydrogen-bond geometry (Å, °)

*D*—H⋯*A*	*D*—H	H⋯*A*	*D*⋯*A*	*D*—H⋯*A*
N1—H1*N*⋯O1^i^	0.86 (4)	1.97 (4)	2.831 (7)	175 (4)
N2—H2*N*⋯O3^ii^	0.86 (4)	2.18 (4)	2.920 (6)	145 (4)
N3—H3*N*⋯O3^ii^	0.86 (6)	2.04 (6)	2.822 (8)	152 (4)

Symmetry codes: (i) 
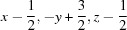
; (ii) 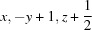.

**Fig. 1.**
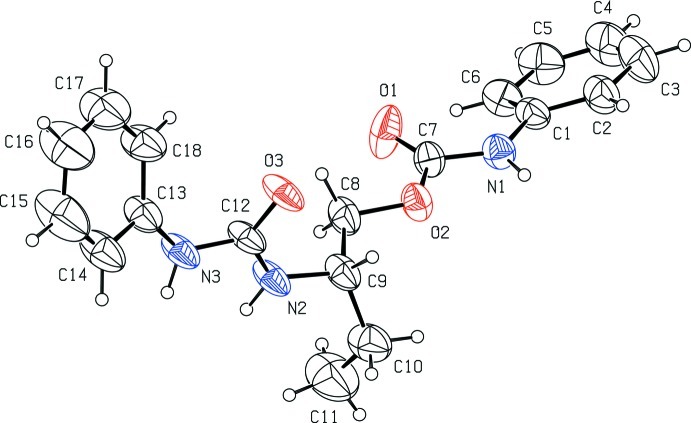
View of (I) with the atom numbering scheme. Displacement ellipsoids for non-H atoms are drawn at the 30% probability level.

**Fig. 2.**
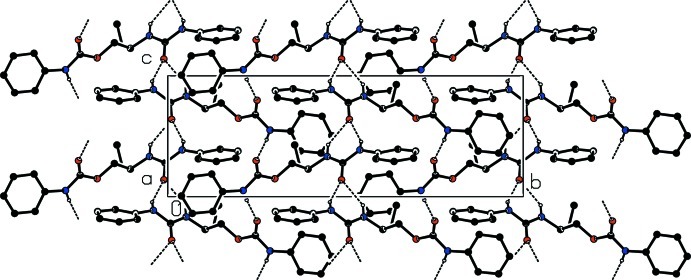
The crystal packing and hydrogen bonding of (I) down the a axis. H atoms not involved in hydrogen bonds have been omitted for clarity.

## supplementary crystallographic information

### Comment

Carbamides and carbamates are great classes of organic compounds due to their incorporations in many of bioactive structures. Carbamides, such as *N*-phenyl-*N*'-(2-chloroethyl)ureas (CEUs) and benzoylureas (BUs) show good anticancer activity, and these compounds have mainly been proved to be tubulin ligands that inhibit the polymerization of tubulin (Li *et al.*, 2009; Gisbert *et al.*, 2004). Carbamates such as aldicarb, carbofuran (Furadan), carbaryl (Sevin), ethienocarb, and fenobucarb are widely used as active pesticides (Metcalf, 2002). Carbamates used also in drug design of anticancer drugs (Ray & Chaturvedi, 2004) and in polymer industry such as polyureathanes. In view of such important applications, we herein report the synthesis and crystal structure of the title compound (I) having both functions of carbamide and carbamate groups.

In (I), (Fig. 1), the orientation of the C1–C6 phenyl ring with respect to the other C13–C18 phenyl ring of the molecule is almost normal by a dihedral angle of 86.3 (5)°. The N1–C7–O2–C8, O2–C8–C9–N2, C9–N2–C12–N3 and C8–C9–C10–C11 torsion angles are 179.8 (5), 169.9 (4), -177.1 (6) and -63.7 (8)°, respectively. The values of the bond lengths and bond angles are consistent with a related structure (Ghalib *et al.*, 2010).

In the crystal, adjacent molecules are interconnected by N—H···O hydrogen bonds (Table 1) into a chain-like structure along the *c* axis generating parallel C(4) and *R*^2^_1_(6) ring motifs (Fig. 2; Bernstein *et al.*, 1995).

### Experimental

The title compound was obtained as a biproduct from a reaction mixture of 89 mg (1 mmol) 2-aminobutan-1-ol, 119 mg (1 mmol) phenylisocyanate and 93 mg (1 mmol) chloroacetone in 50 ml e thanol in presence of few drops of TEA. The reaction mixture was refluxed for 4 h then left to cool at ambient temperature. The solid that formed was decanted washed by ethanol and dried by filteration then recrystallized from acetone. *M*,p. 427 K. Colourless crystals suitable for X-ray diffraction were grown from acetone solution of (1) over 3 days at room temperature. *M*.p. 469 K; 91% yield. IR: spectrum, cm^-1^: 1593 (C=C), 1635 (C=O urea), 1704 (C=O carbamate), 2936–2969 (CH-aliphatic), 3088 (CH-aromatic). ^1^H-NMR, p.p.m., (DMSO); 0.93 t (3*H*, CH2—CH3), 1.5 m (2*H*, CH—CH2—CH3), 3.8 m (1*H*, CH2—CH—CH2), 4.0 d (2*H*, O—CH2—CH), 6.1 s (CH—NH—CO), 6.9–7.4 (m, 10H, Ar), 8.4 s (NH—CO—NH—Ph), 9.6 s (O—CO—NH—Ph). ^13^C-NMR spectrum, d, p.p.m. (DMSO-d): 10.2 (CH3, CH3CH2), 24 (CH2, CH3CH2), 50.2 (CH, CH2—CH—CH2), 66.2 (CH2, O—CH2—CH), 117–128 (10 CH, Ar), 153, 140 (2 C, Ar), 153 (HNCONH, urea), 172 (carbamte).

### Refinement

Carbon-bound H-atoms were placed geometrically (C—H = 0.93 to 0.98 Å) and were refined using a riding model with *U*_iso_=1.2 or 1.5*U*_eq_(C). The N-bound H atom was located from a difference map and refined freely with the distance restraint N—H = 0.86 ± 0.02 Å. In the absence of significant anomalous scattering, the absolute configuration could not be reliably determined so the Friedel pairs were merged and any references to the Flack parameter [-0.3 (5)] were removed.

### Crystal data

**Table:**

C_18_H_21_N_3_O_3_	*F*(000) = 696
*M**_r_* = 327.38	*D*_x_ = 1.198 Mg m^−^^3^
Monoclinic, *C**c*	Cu *K*α radiation, λ = 1.54184 Å
Hall symbol: C -2yc	Cell parameters from 838 reflections
*a* = 10.722 (5) Å	θ = 4.0–72.5°
*b* = 22.297 (3) Å	µ = 0.68 mm^−^^1^
*c* = 9.109 (3) Å	*T* = 293 K
β = 123.570 (6)°	Plate, colourless
*V* = 1814.5 (11) Å^3^	0.14 × 0.12 × 0.07 mm
*Z* = 4	

### Data collection

**Table:**

Oxford Diffraction Xcalibur (Sapphire3, Gemini) diffractometer	1264 reflections with *I* > 2σ(*I*)
Radiation source: Enhance (Cu) X-ray Source	*R*_int_ = 0.024
Graphite monochromator	θ_max_ = 72.7°, θ_min_ = 4.0°
Detector resolution: 16.3280 pixels mm^-1^	*h* = −10→13
ω scans	*k* = −26→27
2915 measured reflections	*l* = −11→8
1676 independent reflections	

### Refinement

**Table:**

Refinement on *F*^2^	Secondary atom site location: difference Fourier map
Least-squares matrix: full	Hydrogen site location: inferred from neighbouring sites
*R*[*F*^2^ > 2σ(*F*^2^)] = 0.052	H atoms treated by a mixture of independent and constrained refinement
*wR*(*F*^2^) = 0.155	*w* = 1/[σ^2^(*F*_o_^2^) + (0.0822*P*)^2^] where *P* = (*F*_o_^2^ + 2*F*_c_^2^)/3
*S* = 1.05	(Δ/σ)_max_ < 0.001
1676 reflections	Δρ_max_ = 0.22 e Å^−^^3^
231 parameters	Δρ_min_ = −0.22 e Å^−^^3^
5 restraints	Extinction correction: *SHELXL97* (Sheldrick, 2008), FC^*^=KFC[1+0.001XFC^2^Λ^3^/SIN(2Θ)]^-1/4^
Primary atom site location: structure-invariant direct methods	Extinction coefficient: 0.0019 (5)

### Special details

**Table:**

Geometry. Bond distances, angles *etc*. have been calculated using the rounded fractional coordinates. All su's are estimated from the variances of the (full) variance-covariance matrix. The cell e.s.d.'s are taken into account in the estimation of distances, angles and torsion angles
Refinement. Refinement on *F*^2^ for ALL reflections except those flagged by the user for potential systematic errors. Weighted *R*-factors *wR* and all goodnesses of fit *S* are based on *F*^2^, conventional *R*-factors *R* are based on *F*, with *F* set to zero for negative *F*^2^. The observed criterion of *F*^2^ > σ(*F*^2^) is used only for calculating -*R*-factor-obs *etc*. and is not relevant to the choice of reflections for refinement. *R*-factors based on *F*^2^ are statistically about twice as large as those based on *F*, and *R*-factors based on ALL data will be even larger.

### Fractional atomic coordinates and isotropic or equivalent isotropic displacement parameters (Å^2^)

**Table:**

	*x*	*y*	*z*	*U*_iso_*/*U*_eq_	
O1	0.2114 (5)	0.75128 (17)	0.7981 (6)	0.1177 (16)	
O2	−0.0009 (4)	0.69881 (13)	0.6486 (4)	0.0788 (10)	
O3	0.0605 (5)	0.51102 (14)	0.6398 (5)	0.0941 (16)	
N1	0.0064 (5)	0.78786 (17)	0.5510 (5)	0.0734 (14)	
N2	0.0156 (6)	0.55146 (15)	0.8323 (5)	0.0847 (18)	
N3	0.1249 (6)	0.45933 (16)	0.8872 (6)	0.0886 (18)	
C1	0.0557 (6)	0.84566 (19)	0.5415 (6)	0.0722 (16)	
C2	−0.0217 (7)	0.8733 (2)	0.3778 (8)	0.092 (2)	
C3	0.0180 (10)	0.9309 (3)	0.3609 (11)	0.124 (3)	
C4	0.1309 (10)	0.9615 (3)	0.5060 (12)	0.123 (3)	
C5	0.2039 (8)	0.9341 (3)	0.6652 (11)	0.110 (3)	
C6	0.1700 (7)	0.8764 (2)	0.6871 (8)	0.088 (2)	
C7	0.0846 (6)	0.74643 (18)	0.6781 (6)	0.0704 (16)	
C8	0.0670 (7)	0.6502 (2)	0.7745 (7)	0.0822 (18)	
C9	−0.0553 (6)	0.60516 (19)	0.7281 (6)	0.0797 (18)	
C10	−0.1753 (9)	0.6283 (3)	0.7544 (10)	0.111 (3)	
C11	−0.1308 (16)	0.6430 (4)	0.9279 (16)	0.182 (6)	
C12	0.0681 (6)	0.50769 (18)	0.7795 (6)	0.0771 (18)	
C13	0.1897 (7)	0.4088 (2)	0.8611 (7)	0.088 (2)	
C14	0.1571 (11)	0.3526 (2)	0.8931 (10)	0.120 (3)	
C15	0.2259 (17)	0.3026 (3)	0.8774 (17)	0.180 (6)	
C16	0.3162 (16)	0.3086 (4)	0.8189 (17)	0.184 (7)	
C17	0.3521 (12)	0.3633 (3)	0.7913 (13)	0.151 (5)	
C18	0.2891 (9)	0.4141 (3)	0.8129 (10)	0.111 (3)	
H1N	−0.085 (3)	0.778 (2)	0.472 (5)	0.087 (17)*	
H2	−0.09950	0.85340	0.28010	0.1110*	
H2N	0.020 (5)	0.549 (2)	0.929 (4)	0.072 (13)*	
H3	−0.03200	0.94910	0.25070	0.1480*	
H3N	0.092 (6)	0.456 (2)	0.954 (6)	0.087 (15)*	
H4	0.15640	1.00020	0.49460	0.1470*	
H5	0.27940	0.95480	0.76300	0.1320*	
H6	0.22280	0.85830	0.79760	0.1050*	
H8A	0.11030	0.66520	0.89320	0.0990*	
H8B	0.14570	0.63160	0.76810	0.0990*	
H9	−0.10400	0.59450	0.60360	0.0950*	
H10A	−0.21960	0.66380	0.68200	0.1330*	
H10B	−0.25340	0.59820	0.70980	0.1330*	
H11A	−0.20880	0.66540	0.92430	0.2720*	
H11B	−0.04100	0.66660	0.98310	0.2720*	
H11C	−0.11230	0.60680	0.99410	0.2720*	
H14	0.08890	0.34810	0.92510	0.1440*	
H15	0.20950	0.26490	0.90750	0.2150*	
H16	0.35410	0.27460	0.79740	0.2200*	
H17	0.41930	0.36720	0.75760	0.1800*	
H18	0.31480	0.45190	0.79440	0.1320*	

### Atomic displacement parameters (Å^2^)

**Table:**

	*U*^11^	*U*^22^	*U*^33^	*U*^12^	*U*^13^	*U*^23^
O1	0.073 (3)	0.078 (2)	0.119 (3)	−0.0025 (17)	0.001 (3)	0.014 (2)
O2	0.086 (2)	0.0655 (16)	0.0737 (18)	−0.0028 (14)	0.0372 (17)	0.0139 (14)
O3	0.176 (4)	0.0685 (17)	0.094 (2)	0.005 (2)	0.110 (3)	0.0044 (16)
N1	0.077 (3)	0.0649 (19)	0.074 (2)	−0.0062 (17)	0.039 (2)	0.0022 (17)
N2	0.159 (4)	0.0572 (18)	0.085 (3)	0.016 (2)	0.097 (3)	0.0076 (18)
N3	0.161 (4)	0.0644 (19)	0.095 (3)	0.021 (2)	0.105 (3)	0.0121 (18)
C1	0.089 (3)	0.060 (2)	0.085 (3)	−0.001 (2)	0.059 (3)	0.006 (2)
C2	0.110 (4)	0.074 (3)	0.099 (4)	0.002 (3)	0.062 (3)	0.014 (3)
C3	0.177 (7)	0.081 (3)	0.127 (5)	0.003 (4)	0.092 (6)	0.027 (4)
C4	0.161 (7)	0.070 (3)	0.159 (7)	−0.015 (4)	0.102 (6)	0.009 (4)
C5	0.131 (5)	0.071 (3)	0.141 (6)	−0.023 (3)	0.083 (5)	−0.016 (4)
C6	0.101 (4)	0.072 (3)	0.097 (4)	−0.016 (3)	0.059 (3)	−0.010 (3)
C7	0.081 (3)	0.052 (2)	0.076 (3)	0.0030 (19)	0.042 (3)	0.0076 (18)
C8	0.109 (4)	0.062 (2)	0.081 (3)	0.010 (2)	0.056 (3)	0.016 (2)
C9	0.124 (4)	0.057 (2)	0.081 (3)	0.007 (2)	0.071 (3)	0.0054 (19)
C10	0.150 (6)	0.079 (3)	0.145 (6)	0.008 (4)	0.108 (5)	0.010 (3)
C11	0.284 (13)	0.146 (8)	0.215 (10)	0.065 (8)	0.201 (11)	0.031 (7)
C12	0.135 (4)	0.053 (2)	0.086 (3)	0.003 (2)	0.088 (3)	0.0014 (19)
C13	0.144 (5)	0.066 (2)	0.096 (3)	0.019 (3)	0.092 (4)	0.006 (2)
C14	0.218 (8)	0.068 (3)	0.147 (5)	0.020 (4)	0.146 (6)	0.015 (3)
C15	0.345 (14)	0.071 (4)	0.246 (11)	0.052 (6)	0.241 (12)	0.034 (5)
C16	0.322 (15)	0.093 (5)	0.268 (13)	0.075 (7)	0.246 (13)	0.041 (6)
C17	0.228 (10)	0.113 (5)	0.204 (9)	0.051 (6)	0.178 (9)	0.020 (5)
C18	0.165 (6)	0.085 (3)	0.141 (5)	0.015 (4)	0.122 (5)	0.002 (3)

### Geometric parameters (Å, º)

**Table:**

O1—C7	1.188 (8)	C13—C14	1.375 (8)
O2—C7	1.330 (6)	C14—C15	1.387 (16)
O2—C8	1.448 (6)	C15—C16	1.35 (3)
O3—C12	1.232 (7)	C16—C17	1.344 (14)
N1—C1	1.414 (6)	C17—C18	1.387 (13)
N1—C7	1.350 (6)	C2—H2	0.9300
N2—C9	1.454 (6)	C3—H3	0.9300
N2—C12	1.342 (7)	C4—H4	0.9300
N3—C12	1.355 (6)	C5—H5	0.9300
N3—C13	1.411 (8)	C6—H6	0.9300
N1—H1N	0.86 (4)	C8—H8A	0.9700
N2—H2N	0.86 (4)	C8—H8B	0.9700
N3—H3N	0.86 (6)	C9—H9	0.9800
C1—C6	1.390 (8)	C10—H10A	0.9700
C1—C2	1.387 (7)	C10—H10B	0.9700
C2—C3	1.388 (9)	C11—H11A	0.9600
C3—C4	1.381 (12)	C11—H11B	0.9600
C4—C5	1.354 (12)	C11—H11C	0.9600
C5—C6	1.381 (9)	C14—H14	0.9300
C8—C9	1.515 (9)	C15—H15	0.9300
C9—C10	1.523 (12)	C16—H16	0.9300
C10—C11	1.414 (15)	C17—H17	0.9300
C13—C18	1.364 (13)	C18—H18	0.9300
			
C7—O2—C8	116.7 (4)	C3—C2—H2	120.00
C1—N1—C7	126.9 (5)	C2—C3—H3	120.00
C9—N2—C12	122.5 (4)	C4—C3—H3	120.00
C12—N3—C13	125.4 (5)	C3—C4—H4	121.00
C7—N1—H1N	115 (3)	C5—C4—H4	121.00
C1—N1—H1N	118 (3)	C4—C5—H5	119.00
C9—N2—H2N	116 (3)	C6—C5—H5	119.00
C12—N2—H2N	122 (3)	C1—C6—H6	120.00
C13—N3—H3N	119 (3)	C5—C6—H6	121.00
C12—N3—H3N	113 (3)	O2—C8—H8A	110.00
N1—C1—C6	123.6 (4)	O2—C8—H8B	110.00
C2—C1—C6	119.6 (5)	C9—C8—H8A	110.00
N1—C1—C2	116.7 (5)	C9—C8—H8B	110.00
C1—C2—C3	119.5 (6)	H8A—C8—H8B	109.00
C2—C3—C4	120.7 (7)	N2—C9—H9	108.00
C3—C4—C5	118.9 (7)	C8—C9—H9	108.00
C4—C5—C6	122.2 (7)	C10—C9—H9	108.00
C1—C6—C5	119.0 (6)	C9—C10—H10A	108.00
O2—C7—N1	110.0 (5)	C9—C10—H10B	108.00
O1—C7—O2	124.7 (4)	C11—C10—H10A	108.00
O1—C7—N1	125.4 (5)	C11—C10—H10B	108.00
O2—C8—C9	107.3 (5)	H10A—C10—H10B	107.00
N2—C9—C10	111.0 (5)	C10—C11—H11A	109.00
C8—C9—C10	114.1 (5)	C10—C11—H11B	109.00
N2—C9—C8	107.7 (5)	C10—C11—H11C	109.00
C9—C10—C11	117.5 (10)	H11A—C11—H11B	110.00
N2—C12—N3	115.4 (5)	H11A—C11—H11C	109.00
O3—C12—N3	122.9 (5)	H11B—C11—H11C	109.00
O3—C12—N2	121.7 (4)	C13—C14—H14	120.00
N3—C13—C14	119.0 (8)	C15—C14—H14	120.00
N3—C13—C18	122.0 (5)	C14—C15—H15	120.00
C14—C13—C18	118.9 (7)	C16—C15—H15	120.00
C13—C14—C15	119.9 (12)	C15—C16—H16	120.00
C14—C15—C16	120.1 (9)	C17—C16—H16	120.00
C15—C16—C17	120.5 (12)	C16—C17—H17	120.00
C16—C17—C18	120.1 (13)	C18—C17—H17	120.00
C13—C18—C17	120.2 (8)	C13—C18—H18	120.00
C1—C2—H2	120.00	C17—C18—H18	120.00
			
C7—O2—C8—C9	172.8 (5)	C6—C1—C2—C3	1.3 (12)
C8—O2—C7—N1	−179.8 (5)	C1—C2—C3—C4	−1.7 (15)
C8—O2—C7—O1	0.8 (9)	C2—C3—C4—C5	0.9 (17)
C1—N1—C7—O1	−4.2 (10)	C3—C4—C5—C6	0.5 (16)
C7—N1—C1—C6	−22.7 (10)	C4—C5—C6—C1	−1.0 (14)
C1—N1—C7—O2	176.3 (5)	O2—C8—C9—C10	−66.4 (6)
C7—N1—C1—C2	160.8 (6)	O2—C8—C9—N2	169.9 (4)
C12—N2—C9—C8	−86.4 (6)	C8—C9—C10—C11	−63.7 (8)
C9—N2—C12—N3	−177.1 (6)	N2—C9—C10—C11	58.2 (8)
C12—N2—C9—C10	148.1 (6)	N3—C13—C14—C15	−176.5 (8)
C9—N2—C12—O3	0.9 (10)	C18—C13—C14—C15	0.5 (12)
C13—N3—C12—N2	−178.2 (6)	N3—C13—C18—C17	179.0 (7)
C13—N3—C12—O3	3.9 (10)	C14—C13—C18—C17	2.1 (11)
C12—N3—C13—C18	44.7 (10)	C13—C14—C15—C16	−4.7 (17)
C12—N3—C13—C14	−138.4 (7)	C14—C15—C16—C17	6 (2)
N1—C1—C6—C5	−176.3 (7)	C15—C16—C17—C18	−3.9 (19)
C2—C1—C6—C5	0.1 (12)	C16—C17—C18—C13	−0.4 (14)
N1—C1—C2—C3	177.9 (8)		

### Hydrogen-bond geometry (Å, º)

**Table:**

*D*—H···*A*	*D*—H	H···*A*	*D*···*A*	*D*—H···*A*
N1—H1*N*···O1^i^	0.86 (4)	1.97 (4)	2.831 (7)	175 (4)
N2—H2*N*···O3^ii^	0.86 (4)	2.18 (4)	2.920 (6)	145 (4)
N3—H3*N*···O3^ii^	0.86 (6)	2.04 (6)	2.822 (8)	152 (4)
C6—H6···O1	0.93	2.39	2.917 (6)	116

Symmetry codes: (i) *x*−1/2, −*y*+3/2, *z*−1/2; (ii) *x*, −*y*+1, *z*+1/2.
